# Mapping Global Trends and Research Hotspots in Postoperative Depression: A Bibliometric Analysis

**DOI:** 10.1155/bn/5578017

**Published:** 2026-08-06

**Authors:** Ting Li, Liqin Zhao, Min Li, Hailong Zhang

**Affiliations:** ^1^ Department of Anesthesiology, Beijing Luhe Hospital, Capital Medical University, Beijing, China, ccmu.edu.cn; ^2^ Department of Anesthesiology, Beijing Ditan Hospital, Capital Medical University, Beijing, China, ccmu.edu.cn

**Keywords:** bibliometric analysis, CiteSpace, global trends, postoperative depression, VOSviewer

## Abstract

**Background:**

This bibliometric analysis is aimed at mapping global trends and research hotspots in postoperative depression, providing insights into the evolution and development of research in this field.

**Methods:**

A comprehensive search was conducted in the Web of Science Core Collection database, focusing on literature from 1994 to 2024. Data extraction and visualization were performed using VOSviewer (Version 1.6.20), CiteSpace (Version 6.3.R1), and R (Version 4.3.3).

**Results:**

This study encompassed 687 scholarly articles from 54 countries. The United States contributed the largest number of articles, followed by China and the United Kingdom. High‐output institutions from the United States include the University of California System and Harvard University. The *Journal of Psychosomatic Research* and *Epilepsy* were among the top journals by h‐index. Aalto Timo, Airaksinen Olavi, and Sinikallio Sanna were among the most productive authors. High‐frequency keywords included “quality‐of‐life,” “anxiety,” and “symptoms.” Recent burst keywords “risk,” “elderly patients,” and “double blind” indicate emerging trends in the field, pointing toward an increased interest in identifying factors that may precipitate postoperative depression, the unique vulnerabilities of older adults, and the methodological rigor of double‐blind studies in assessing interventions.

**Conclusion:**

The research hotspots and trends in postoperative depression are centered around the impact of depression on surgical outcomes, the role of anxiety, and quality of life assessments. This study reveals a growing interest in predictors of postoperative depression and the exploration of novel therapeutic interventions, indicating an evolving and dynamic research area.

## 1. Introduction

Depression, a prevalent and debilitating mood disorder, is associated with substantial individual suffering and societal costs. The condition is characterized by a persistent low mood, loss of interest or pleasure, and a range of physical and psychological symptoms that significantly impair daily functioning [1]. The global landscape of depressive symptoms from 2001 to 2020 reveals a point prevalence of 34%, with major depressive disorder (MDD) and dysthymia affecting 8% and 4% of the population, respectively [2]. Despite the availability of pharmacological and psychotherapeutic interventions, challenges in diagnosis and treatment persist, often due to the complexity of the disorder, stigma, and limited access to healthcare services [3]. In the clinical context, depression is particularly significant in the perioperative period, where it can exacerbate postoperative outcomes and complicate recovery [4]. Postoperative depression, in particular, is a critical concern, as it can lead to prolonged hospital stays and diminished quality of life.

Postoperative depression is characterized by a spectrum of mood disturbances that can range from subclinical symptoms to MDD episodes, with potential long‐term implications for patient recovery and quality of life [5, 6]. The postoperative depression reveals a complex interplay of genetic, psychological, and environmental factors, contributing to its variable presentation and diagnosis. The pathophysiology underlying this condition is not yet fully elucidated but is thought to involve neurochemical imbalances, particularly in the monoamine neurotransmitter systems, as well as alterations in the hypothalamic–pituitary–adrenal (HPA) axis [7, 8]. Risk factors for developing postoperative depression are multifaceted and include preexisting mental health conditions, gender, age, surgical trauma, and the presence of chronic medical comorbidities [9, 10]. The clinical manifestations can be subtle, often overshadowed by the physical symptoms of surgery, leading to underdiagnosis and undertreatment. Furthermore, there is a growing recognition of the bidirectional relationship between depression and surgical outcomes, where depression can both result from and contribute to adverse postoperative events [11, 12]. This underscores the imperative for a comprehensive understanding of postoperative depression to inform the development of effective screening, prevention, and treatment strategies.

Bibliometric analysis is a quantitative research method that involves the statistical analysis of written publications to extract and map the intellectual structures of a given field [13]. It employs various metrics, such as citation counts and cocitation frequencies, to identify trends, patterns, and works within academic disciplines, thereby providing insights into the evolution and impact of research over time [13]. A recent bibliometric analysis emphasizes the pivotal role of brain‐derived neurotrophic factor (BDNF) in the pathogenesis and therapeutic mechanisms of depression [14]. Furthermore, the work by Li et al. [15] emphasizes the growing focus on Alzheimer′s and depression, highlighting themes of disease mechanisms, early detection, enhancing quality of life, and managing neuropsychiatric symptoms. In addition, recent bibliometric studies have revealed significant trends in postoperative pain management after cesarean sections, postoperative delirium, and the interplay between postoperative pain and sleep disorders, as evidenced by the growing body of literature and the emergence of new research foci [16–18]. However, to date, there has been a notable absence of bibliometric analysis specifically targeting postoperative depression.

This study is aimed at filling the research gap by conducting a bibliometric analysis to map the global trends and identifying key research areas in postoperative depression. It seeks to uncover the intellectual structure and evolution of this field, highlighting highly cited studies and authors, and predicting future directions for research.

## 2. Materials and Methods

### 2.1. Literature Review and Selection Process

The Web of Science Core Collection (WoSCC) database, specifically the Science Citation Index Expanded (SCIE) and Social Sciences Citation Index (SSCI), was used as the primary data source for this bibliometric analysis due to its wide disciplinary coverage, standardized indexing, and reliability for citation analysis [13, 14]. The literature search was conducted on November 21, 2024, covering publications from January 1994 to November 2024, thereby providing three decades of research context. In this study, “postoperative depression” was operationally defined as any depressive symptomatology (including MDD, subclinical depression, and mood disturbance) that occurs following surgical interventions.

**Figure 1:**
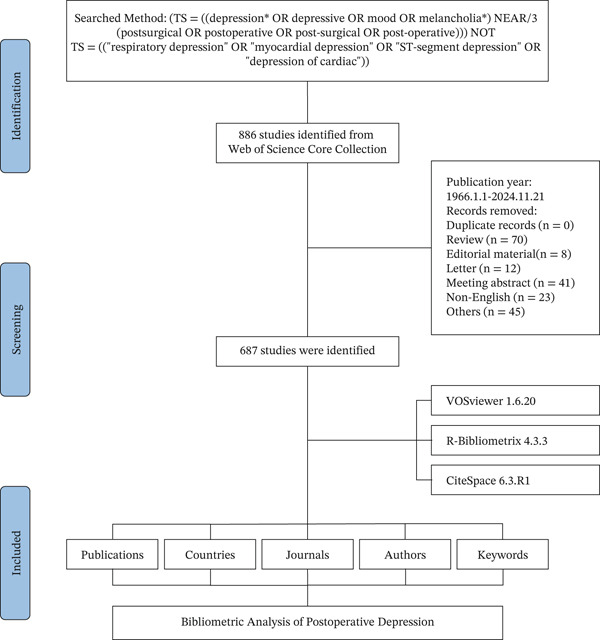
Flowchart of the literature screening process, detailing the methodical approach to identify and select relevant studies for analysis.

Following previously published bibliometric and meta‐analytic studies examining perioperative affective disorders [16–18], the search strategy was designed to capture depressive or mood‐related outcomes after surgery while excluding unrelated physiological “depression” terms, such as respiratory depression, myocardial depression, ST‐segment depression, or depression of cardiac function. This approach ensures a comprehensive yet conceptually focused retrieval of the postoperative depression outcomes. The final search strategy applied in the WoSCC was [19, 20]: (TS = ((depression∗ OR depressive OR mood OR melancholia∗) NEAR/3 (postsurgical OR postoperative OR post‐surgical OR post‐operative))) NOT TS = ((“respiratory depression” OR “myocardial depression” OR “ST‐segment depression” OR “depression of cardiac”)). This retrieval logic has been shown in prior bibliometric research to capture the vast majority of publications relevant to postoperative depression while maintaining high conceptual specificity [15, 21]. All inclusion/exclusion criteria and filtering steps are summarized in the PRISMA‐like flow diagram (Figure 1), ensuring transparency and reproducibility. The initial search identified 886 records after limiting the database to SCIE and SSCI indices. Only articles written in English were considered, ensuring a uniformity of language and facilitating a comprehensive analysis. To facilitate a thorough bibliometric analysis, the full record and cited references, along with plain text, were exported for each article. This approach allowed for the extraction of a wealth of information, including country/region, institution, journal, publication/citation metrics, author details, and keywords, providing a multidimensional view of the research landscape.

### 2.2. Data Analysis and Visualization

We employed a triad of analytical tools to conduct a comprehensive bibliometric analysis of the academic data. These tools included VOSviewer (Version 1.6.20), CiteSpace (Version 6.3.R1), and R (Version 4.3.3). VOSviewer, a versatile software tool, was pivotal in mapping complex collaboration and relationship networks within the academic domain [22]. By employing VOSviewer, we were able to delve into the intricate connections between authors, institutions, and publications. For a deeper understanding of emerging trends and research hotspots within the field, we utilized VOSviewer for keyword co‐occurrence analysis and CiteSpace for keyword burst detection. CiteSpace (Version 6.3.R1) was employed for keyword burst detection and timeline visualization. The parameters were set with a time slicing feature spanning from January 1994 to November 2024, with a 1‐year interval for each slice. The node types were designated as keywords. When nodes were set to keywords, the threshold was set to the top five keywords per slice, and pruning was conducted using the Pathfinder network merging algorithm. These parameter settings were used to generate a timeline map of keywords in the field of postoperative depression research. In the visualizations, the size of nodes represented the frequency or impact of the associated entities, such as the number of citations for an author or the occurrence of a keyword. The color coding was used to distinguish between different clusters and categories, such as various research topics or time periods. The connections between nodes, or edges, signified relationships such as coauthorship or cocitation, with the thickness of the lines indicating the strength of the relationship. Additionally, R was utilized for statistical analysis and data visualization. With R, we performed advanced statistical tests and created custom visualizations that provided further insights into the data. Normalized TC was additionally used as a supplementary time‐adjusted citation indicator to reduce the potential time‐dependent bias of raw citation counts.

The h‐index, a measure of both the productivity and citation impact of the publications of a scientist or scholar, was calculated to assess the influence of individual authors [23, 24]. The g‐index extends the h‐index by considering the citation counts, providing a more nuanced view of an author′s citation impact [25]. The m‐index was considered as a time‐adjusted derivative of the h‐index, reflecting citation impact relative to the time span of an author′s publications in the dataset. In addition to these author‐level metrics, the impact factor (IF) of journals and their Journal Citation Reports (JCR) rankings were considered to evaluate the prestige and reach of the publications within our dataset. These metrics are crucial for understanding the broader context of the research field and the significance of the findings presented in the indexed journals. By leveraging these bibliometric tools and metrics, we were able to efficiently organize and analyze the bibliometric data extracted from the literature records.

## 3. Results

### 3.1. Annual Publication Trends in Postoperative Depression Research

**Figure 2:**
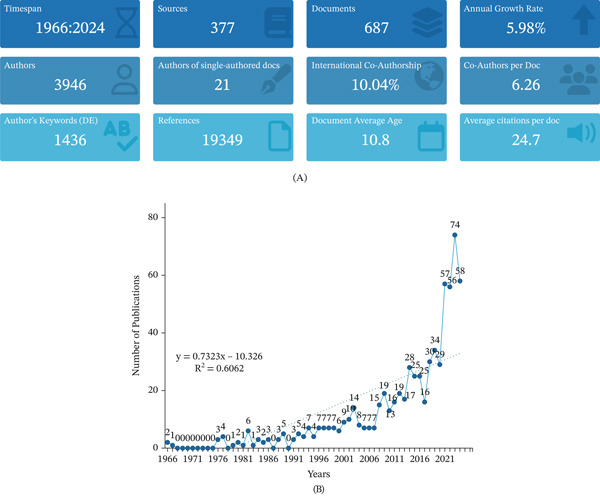
Overview of the analysis, providing a comprehensive summary of the bibliometric analysis conducted on scholarly publications from 1994 to 2024. (A) Bibliometric analysis summary of scholarly publications, showcasing the distribution and trends from 1994 to 2024. (B) Annual number of publications, illustrating the growth and fluctuations in research output over time.

The initial search identified 886 records after limiting the database to SCIE and SSCI indices (Figure 1). After excluding 199 records, including 23 non‐English publications and 176 nonarticle document types (70 reviews, 8 editorials, 12 letters, 41 meeting abstracts, and 45 other document types), 687 articles remained and were included in the final bibliometric analysis. A total of 3946 authors (Figure 2A), from 2175 institutions in 54 countries contributed to the included articles. The 687 articles were published in 377 journals, with 19,349 cumulative citations and an annual growth rate of 5.98%. Annual output increased overall, with a brief decrease to 16 publications in 2017 (Figure 2B) and a maximum of 74 publications in 2023. The fitted trend line was y = 0.7323x − 10.326, with *R* ^2^ = 0.6062, corresponding to an average increase of approximately 0.73 publications per year (slope = 0.7323).

### 3.2. Country/Region Publication Output and Collaboration

**Figure 3:**
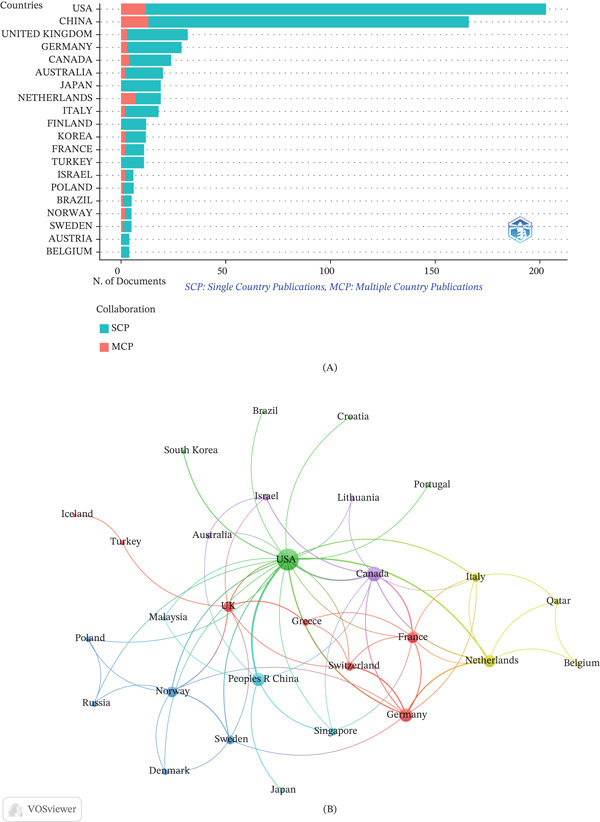
Analysis of countries, highlighting the global contribution to the field, with a focus on the distribution of publications by country. (A) Distribution of corresponding author′s publications by country, offering insights into national research contributions. (B) Visualization map depicting the collaboration among different countries, emphasizing international research partnerships. Each node represents a country/region; node size is proportional to publication output (number of documents in the dataset), and link thickness represents the strength of collaboration (coauthored publications) between countries (i.e., total link strength in VOSviewer).

The bibliometric analysis identified the United States as the leading contributor, with 203 articles (29.5%) and 751 total publications (TP, total number of publications, including single‐country and internationally coauthored papers) (Figure 3A). China followed with 166 articles (24.2%) and 479 TP, whereas the United Kingdom ranked third with 32 articles (4.7%) and 94 TP (Table S1). Canada contributed 24 articles, ranking fifth by output but third by TP = 111. In total citations (TC, total citations received by the included articles), the United States, China, and United Kingdom ranked first (6792), second (1284), and third (1083), respectively. Finland and Australia demonstrated the highest average citations per article (45.4 and 42.6). Among the 39 countries engaged in international collaborations (≥ 2 articles), the United States exhibited the highest total link strength (44), followed by Canada (20) and China (16) (Figure 3B).

### 3.3. Journal Output and Journal Networks

**Figure 4:**
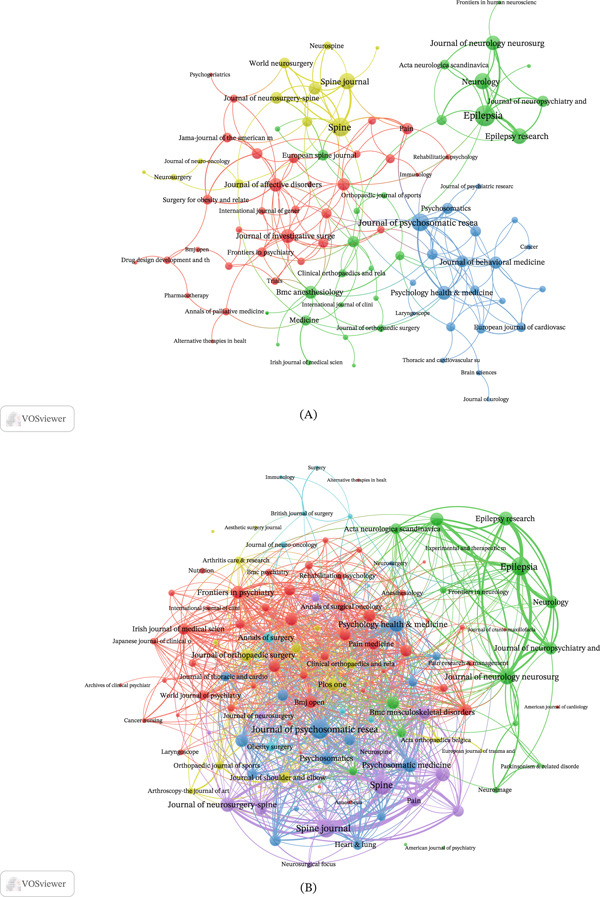
Analysis of journals, examining the impact and reach of various journals in the field. (A) Journal co‐occurrence network based on cited sources, in which two journals are linked when they coappear in the reference list of the same included article. Each node represents a journal; node size is proportional to its occurrence frequency in the dataset, and link thickness reflects co‐occurrence strength (total link strength). (B) Coupling networks, where two journals are linked when articles published in those journals share references. Node size is proportional to the number of documents from each journal in the dataset, and link thickness indicates coupling strength (the number of shared references).

A total of 377 journals published articles on postoperative depression. *Epilepsy* (IF = 6.6, Q1), *Journal of Psychosomatic Research* (IF = 3.5, Q2), and *PLoS One* (IF = 2.9, Q1) ranked highest by h‐index (a citation‐based index reflecting both productivity and citation impact of a journal/author within the dataset) (10, 10, and 8) (Table S2). *Journal of Psychosomatic Research* leads with 11 publications, ranking first in TP, whereas *Epilepsy*, with 10 publications, ranks second in TP. *PLoS One* follows with eight publications, ranking eighth in TP. In terms of TC, *Journal of Psychosomatic Research* ranks second with 287 citations, and *Epilepsy* ranks third with 285 citations. The co‐occurrence network, comprising 92 journals (≥ 2 occurrences), revealed three main citation hubs: *Epilepsy*, *Spine*, and *Journal of Psychosomatic Research*, with total link strengths of 35, 27, and 24, respectively (Figure 4A). In the coupling analysis (120 journals cited together ≥ 2 times), connectivity peaked for *Journal of Psychosomatic Research* (540), *Spine* (525), and *Epilepsia* (473) (Figure 4B).

### 3.4. Author Productivity and Collaboration

**Figure 5:**
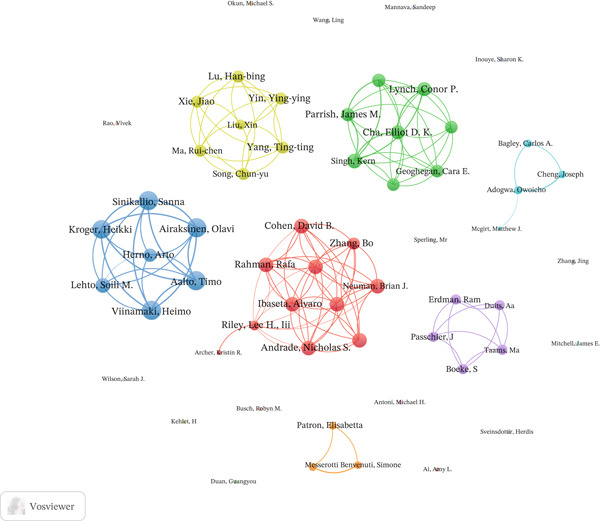
Visualization map depicting the collaboration among different authors, highlighting the collaborative efforts in advancing the field. Each node represents an author; node size is proportional to the author′s publication count in the dataset, and link thickness reflects the strength of collaboration (number of coauthored publications/total link strength).

The leading contributors were Aalto Timo, Airaksinen Olavi, Sinikallio Sanna, and Viinamäki Heimo, each with nine publications, an h − index = 9, and a g − index = 9 (Table S3). They have collectively published 36 articles, with nine publications per author (TP = 9). Their work has garnered a substantial 416 citations. Within the subgroup of authors engaged in international collaborations (≥ 3 articles), Aalto Timo exhibited the greatest link strength (44), followed closely by Airaksinen Olavi and Sinikallio Sanna (44 each) (Figure 5).

### 3.5. Institutional Output and Collaboration

**Figure 6:**
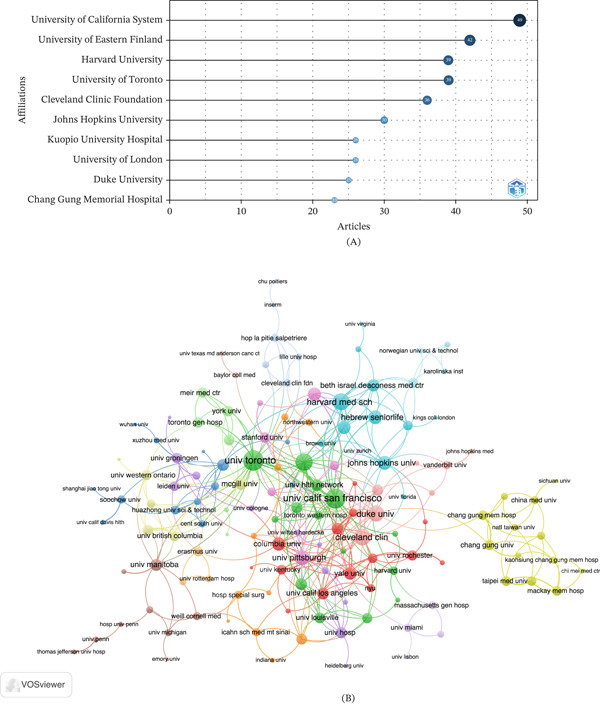
Analysis of institutions, identifying key contributors and their collaborative networks. (A) Top 10 institutions by article count and rank, recognizing the leading centers of research and knowledge production. (B) Visualization map depicting the collaboration among different institutions, showcasing institutional partnerships and research networks. Each node represents an institution; node size is proportional to the institution′s publication output, and link thickness represents collaboration strength based on coauthored publications (total link strength).

Across all 2175 institutions, the University of California System achieved the highest output (49 articles), followed by the University of Eastern Finland (42) and Harvard University (39) (Figure 6A). In collaborative network analysis (≥ 2 joint articles), the University of Toronto demonstrated the highest total link strength (33), with the University of California, San Francisco (32), and University of Washington (24) ranking next (Figure 6B).

### 3.6. Citation Patterns of Highly Cited Publications

The most highly cited publication was “Mental Health Conditions Among Patients Seeking and Undergoing Bariatric Surgery: A Meta‐Analysis” (*JAMA*, 2016, IF = 63.1) with 345 citations [26]. The second cited article was “A Multicentre Study on Suicide Outcomes Following Subthalamic Stimulation for Parkinson′s Disease” (*Brain*, 2008, IF = 10.6; 324 citations) [27]. The third cited work was “Anxiety and Depression in Bariatric Surgery Patients” (*J Affect Disord*, 2011, IF = 4.9; 265 citations) [28]. To reduce the inherent time‐dependent bias of raw citation counts, we additionally considered normalized TC (Table S4). For example, “Influence of Psychological Factors on Postoperative Pain, Mood and Analgesic Requirements.” (*Pain*, 1986) [29] shows a relatively high TC (250) but a lower normalized TC (2.62), whereas “Preoperative PROMIS Scores Predict Postoperative Success in Foot and Ankle Patients.” (Foot & Ankle International, 2016) shows a lower TC (166) but a higher normalized TC (3.85) [30].

### 3.7. Thematic Insights: Keyword Co‐Occurrence and Burst Analysis

**Figure 7:**
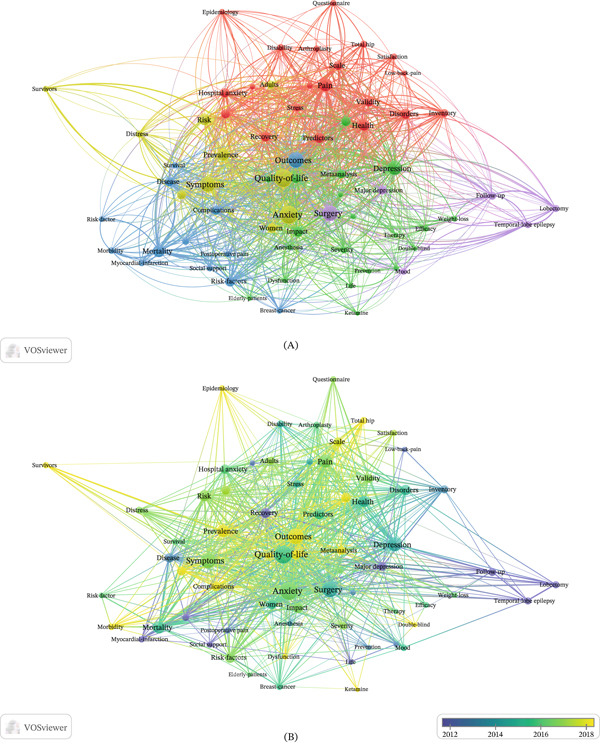
Analysis of keywords, providing a thematic insight into the research landscape. (A) Cluster analysis of co‐occurrence keywords, categorizing the thematic focus areas within the field. (B) Co‐occurrence keyword analysis, where each node′s size corresponds to its occurrence frequency, and node connections indicate co‐occurrence strength. Each node represents a keyword; node size corresponds to its occurrence frequency, and link thickness indicates co‐occurrence strength (how often two keywords appear together).

The network visualization crafted through VOSviewer identified seven distinct clusters of keywords, each representing a thematic focus area within the postoperative depression research domain (Figure 7A and Table S5). Cluster 1: aging and postoperative psychological adaptation. This cluster includes terms such as “adjustment,” “age,” and “elderly‐patients.” Cluster 2: cardiac surgery and mental health outcomes, featuring “artery‐bypass‐surgery,” “cardiac‐surgery,” and “coronary‐artery‐disease.” Cluster 3: pharmacologic and procedure‐related interventions, with keywords “antidepressant,” “bypass graft‐surgery,” and “decompression.” Cluster 4: anxiety‐related comorbidity across surgical specialties, characterized by keywords such as “anxiety,” “arthroplasty,” and “breast‐cancer.” Cluster 5: anesthesia, pain, and neuromodulation‐related topics, encompassing “anesthesia,” “chronic pain,” and “deep‐brain‐stimulation.” Cluster 6: symptom measurement and functional impact, with “behavior,” “depression scale,” and “disability.” Cluster 7: care delivery and sociodemographic context, containing “adults,” “association,” and “care.” In the overall network, “quality‐of‐life,” “anxiety,” “symptoms,” and “outcomes” were the most frequent keywords (occurrences = 112, 101, 76, and 78; total link strength = 467, 448, 357, and 343) (Figure 7B).

**Figure 8:**
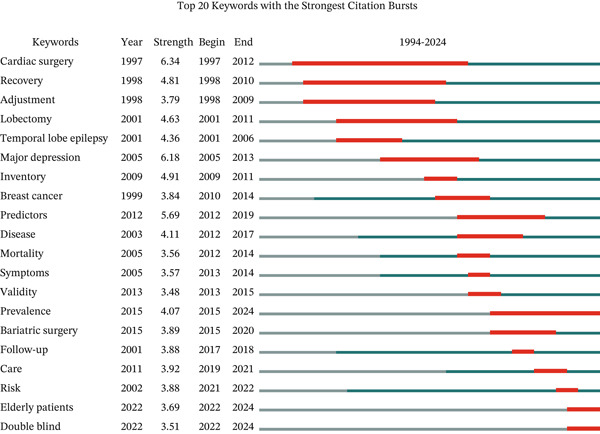
Top 20 keywords with the strongest bursts, identifying the most trending topics in recent research.

The burst keyword analysis illustrates changes in research emphasis over time (Figure 8). Early bursts included “cardiac surgery” (strength = 6.34, 1997–2012), “recovery” (4.81, 1998–2010), and “adjustment” (3.79, 1998–2009). After 2010, bursts shifted to “predictors” (5.69, 2012–2019) and “mortality” (3.56, 2012–2014), with “validity” also emerging (3.48, 2013–2015). More recent bursts were observed for “risk” (3.88, 2021–2024), “elderly patients” (3.69, 2021–2024), and “double blind” (3.51, 2021–2024). In addition, “prevalence” showed a sustained burst from 2015 to 2024 (4.07).

## 4. Discussion

This study offers an in‐depth analysis of the research landscape concerning postoperative depression, revealing a robust and upward trajectory in academic output. The identified growth trend, with a temporary dip in 2017 followed by a sharp increase, culminating in a peak in 2023, underscores a period of significant research advancement and productivity. This surge in scholarly activity likely reflects the heightened focus on the field, potentially driven by emerging research questions, technological advancements, and an increased awareness of the field′s societal impact. The momentum observed in publication volumes is poised to stimulate further exploration and innovation, laying the groundwork for more impactful and transformative research in the foreseeable future.

The United States and China contributed the largest shares of publications, while the United States showed the strongest international collaboration links. Rather than implying intrinsic “strength,” this pattern likely reflects uneven global distribution of research resources, funding capacity, and mature publication infrastructures, which can lead to clustered collaboration structures (“core–periphery” patterns) in which a small set of countries and institutions serve as recurring hubs. Consistent with this, the University of California System and Harvard University appear among the most productive institutions in the dataset [31, 32]. With regard to highly cited work, the 2016 *JAMA* meta‐analysis accumulated 345 citations in the dataset [26]. However, citation volume should be interpreted cautiously because it is shaped by publication year and topic‐specific citation practices. Substantively, this meta‐analysis summarizes mental health conditions among bariatric surgery patients and highlights the clinical relevance of perioperative mental health assessment and follow‐up [26]. Overall, the concentration of output in a few countries/institutions suggests that evidence generation and agenda‐setting in this area may be disproportionately driven by regions with greater research capacity, underscoring the value of broader international participation and multisetting studies.

The author analysis shows that several research teams, such as Aalto Timo, Airaksinen Olavi, Sinikallio Sanna, and Viinamäki Heimo, form stable coauthorship clusters, reflecting sustained research activity on mood symptoms and postoperative outcomes in specific clinical contexts. For example, their prospective work in *Spine* (2011) reported an association between depressive symptoms and less favorable outcomes after lumbar spinal stenosis surgery during follow‐up [33]. Their later work extended follow‐up and suggested that indicators related to life dissatisfaction are linked to longer term outcomes [34]. These studies illustrate how longitudinal clinical cohorts contribute to the evidence base and why perioperative psychosocial assessment can be relevant for postoperative recovery trajectories.

The journal cocitation and coupling networks suggest that postoperative depression research is not confined to a single specialty journal ecosystem. Instead, the field shows a cross‐disciplinary knowledge structure, bridging surgical journals with psychosomatic/psychiatric and behavioral medicine journals. This pattern is consistent with the clinical reality that postoperative depressive symptoms are studied both as mental health outcomes and prognostic/rehabilitation‐related factors affecting surgical recovery. In this context, the *Journal of Psychosomatic Research* functions as a recurrent outlet and citation node connecting mental health assessment with perioperative care. For instance, a recent prospective cohort study in this journal examined depressive and anxiety symptoms in patients with type 2 diabetes and peripheral artery disease undergoing partial foot amputation and reported that higher PHQ‐9 scores were associated with higher 1‐year mortality risk [35]. Collectively, the journal network structure supports the interpretation that postoperative depression is investigated through an integrated surgical–psychological lens, and that collaboration between surgical disciplines and mental health research may be important for advancing patient‐centered perioperative care.

### 4.1. Most Cited Literature in Postoperative Depression

Across the dataset, several publications have accumulated high citation counts, including the *JAMA* meta‐analysis on mental health conditions in bariatric surgery patients [26], the *Brain* study on suicide outcomes following subthalamic stimulation for Parkinson′s disease [27], and a *Journal of Affective Disorders* prospective follow‐up study on anxiety/depression in bariatric surgery patients [28]. Because citation counts are affected by publication year and subfield citation norms, these metrics are best interpreted as indicators of citation visibility rather than definitive measures of scientific or clinical “influence.” Nevertheless, the topics represented by these papers highlight the diversity of surgical populations studied and reinforce the need for longitudinal assessment and appropriate perioperative mental health support.

### 4.2. Thematic Insights Into Postoperative Depression Research

This analysis reveals a multifaceted landscape of postoperative depression research, with seven distinct clusters that encapsulate the thematic foci of the field.

### 4.3. Cluster 1: Geriatric Vulnerability and Perioperative Mental Health

This cluster is dominated by age‐related keywords, suggesting that the literature frequently links older age with postoperative mood‐related outcomes and recovery topics. From a co‐occurrence perspective, the cluster appears to reflect a “risk profile” framing in which patient age is discussed alongside perioperative exposures and subsequent psychological outcomes [36]. Related clinical studies in older populations have examined associations between anesthetic approaches and postoperative depression or anxiety symptoms, which may help explain why anesthesia‐related terms coappear with geriatric keywords in the map [37]. Conceptually, this cluster links closely to Clusters 5–6 (anesthesia/pain management and symptom assessment), because geriatric vulnerability is often operationalized through measured symptoms and influenced by perioperative management strategies.

### 4.4. Cluster 2: Cardiac Surgery Populations and Prognostic Implications

Keywords in this cluster indicate a procedure‐centered research stream in which cardiac surgery terms co‐occur with depression and anxiety constructs, consistent with a sustained focus on cardiovascular surgical populations. In bibliometric terms, the co‐occurrence structure suggests that mental health measures are frequently discussed alongside postoperative outcomes and longer term follow‐up in this setting. This pattern aligns with the emphasis of Takagi et al. [5], who synthesized evidence on perioperative depression or anxiety and postoperative mortality in cardiac surgery patients. This cluster overlaps with Cluster 6 (assessment tools and symptom trajectories) because cardiovascular studies frequently rely on standardized scales and longitudinal follow‐up to relate psychological measures to outcomes

### 4.5. Cluster 3: Pharmacologic and Perioperative Interventions at the Pain–Depression Interface

Keywords including “antidepressant,” together with procedure‐related terms, suggest that a subset of the literature addresses postoperative depression in connection with perioperative management topics, particularly where pain‐related constructs are present [6]. At the map level, the co‐occurrence of drug‐ and surgery‐related terms indicates that depression is often discussed as a potentially modifiable postoperative issue within intervention‐oriented studies, although the bibliometric analysis itself does not evaluate intervention efficacy. Subramanian et al. [21] discussed ketamine′s therapeutic potential across chronic pain and depressive disorders, which provides one plausible explanation for why analgesic or anesthetic pharmacology terms cluster with mood‐related keywords in this field. This cluster connects outward to Clusters 5 (anesthetic techniques) and 4 (psychological predictors of chronic postsurgical pain), forming a broader “pain–mood–recovery” research axis

### 4.6. Cluster 4: Psychological Comorbidity Across Disease‐ and Procedure‐Specific Contexts

The co‐occurrence of “anxiety” with procedure or disease terms such as “arthroplasty” and “breast‐cancer” suggests that postoperative depression research is frequently embedded in broader perioperative psychological symptom discussions across multiple surgical specialties. Within the bibliometric structure, these keywords collectively point to studies that examine psychological factors alongside recovery‐related endpoints, including pain and functional status. Masselin‐Dubois et al. [38] reported psychological predictors of chronic postsurgical pain in knee arthroplasty and breast cancer surgery cohorts, which may contribute to the observed clustering of anxiety‐related terms with procedure‐specific keywords. Rather than indicating uniform effects across surgeries, the cluster should be read as a mapping of where and how these topics coappear in the literature. This cluster bridges to Cluster 7 (broader care and outcomes) by motivating integrated perioperative pathways that incorporate psychological screening and support.

### 4.7. Cluster 5: Anesthesia Strategies and Chronic Pain Management

Keywords such as “anesthesia” and “chronic pain” form a management‐focused cluster that, at the co‐occurrence level, highlights how perioperative technique and pain trajectories are often discussed alongside postoperative psychological outcomes [39]. The keyword structure suggests an interest in perioperative management as a contextual factor within postoperative depression discussions, but it does not establish directionality or mechanisms. Brenna et al. [40] reviewed evidence that some anesthetic agents may have antidepressant‐relevant properties. This cluster links outward to Clusters 3 (pharmacologic interventions) and 6 (assessment), because the evaluation of anesthesia‐related strategies depends on measuring both pain and mood outcomes over time

### 4.8. Cluster 6: Measurement, Screening, and Symptom Trajectories

The co‐occurrence of “behavior” and “depression scale” indicates a methodological theme focused on how postoperative depression and related symptoms are operationalized and tracked in the literature. From a bibliometric viewpoint, this cluster represents the measurement infrastructure that enables comparisons across populations and study designs, as reflected by repeated instrument‐related keywords. Acikel [41] reported changes in postoperative depression and anxiety levels in coronary artery bypass graft patients, illustrating one context in which repeated assessments are used and therefore may contribute to the prominence of scale‐related keywords. This cluster functions as a connector across most other clusters, because geriatric risk (Cluster 1), cardiac prognosis (Cluster 2), and intervention research (Clusters 3 and 5) all require consistent measurement strategies.

### 4.9. Cluster 7: Care Delivery, Sociodemographic Context, and Postoperative Outcomes

Keywords such as “adults,” “association,” and “care” indicate a broad services‐and‐outcomes theme that situates postoperative depression within discussions of patient management and recovery contexts [42]. In the map, these general terms likely function as bridging keywords that co‐occur with multiple procedure‐ and symptom‐focused topics, reflecting a broad, integrative discourse in the field. The overview by Ghoneim and O′Hara [6] summarized associations between depression and postoperative complications, which may contribute to the co‐occurrence between depression‐related keywords and outcome‐oriented terms in this cluster. This cluster ties the entire map together by emphasizing implementation: how screening (Cluster 6) and perioperative management (Clusters 3 and 5) might translate into improved recovery and patient‐centered outcomes across specialties

### 4.10. Prominent Co‐Occurrence Keywords in Postoperative Depression Research

This bibliometric analysis identifies high‐frequency co‐occurrence keywords including “quality‐of‐life,” “anxiety,” “symptoms,” “outcomes,” and “pain,” indicating that postoperative depression research is frequently framed around patient‐reported symptom burden and functional recovery rather than depression as an isolated psychiatric diagnosis. Clinically, the repeated co‐occurrence of “pain,” “anxiety,” and “quality‐of‐life” suggests a consistent emphasis on the pain–sleep–function–mood pathway in postoperative care, in which uncontrolled pain and distress can amplify depressive symptoms and impair rehabilitation. For example, Acikel et al. [41] reported increased depression and anxiety following coronary artery bypass graft surgery, and Depolli et al. [43] observed postoperative improvements in quality of life and symptom measures after thyroidectomy, although not all domains showed direct linear correlations, supporting the view that postoperative mental health and quality‐of‐life trajectories may vary by procedure type, baseline status, and follow‐up duration.

In addition to symptom and quality‐of‐life outcomes, the co‐occurrence of “outcomes” and “pain” highlights that many studies evaluate postoperative depression in relation to prognostic endpoints and perioperative management strategies. Niu et al.′s [44] meta‐analysis summarized evidence on esketamine and suggested potential benefits for depressive symptoms and quality of life, reflecting the field′s growing interest in intervention‐oriented research (pharmacologic strategies) alongside observational risk profiling. Finally, Hazir et al. [45] emphasized in pediatric urology that baseline patient factors and parental psychological status can be strongly associated with postoperative quality of life, illustrating that postoperative mental health is often embedded in a broader family/social context, particularly in pediatrics. Collectively, these keyword patterns support a clinical interpretation that postoperative depression research is currently oriented toward risk recognition and outcome evaluation across diverse surgical populations, with increasing attention to how perioperative interventions and psychosocial context shape recovery.

### 4.11. Burst Keywords Reflecting Research Trends in Postoperative Depression

To address the temporal dimension, we interpret keyword bursts and related hotspot areas as reflecting a staged evolution of clinical attention and research paradigms.

### 4.12. Stage 1: Early Exploration (Approximately Late 1990s–2012): Procedure‐Centered Observations and Recovery/Adjustment

In the early phase, burst terms such as “cardiac surgery” (1997–2012) indicate that postoperative depression was initially investigated most intensively in high‐risk settings where adverse outcomes are common and follow‐up is relatively standardized. Clinically, cardiac surgery cohorts offered clear endpoints (e.g., complications, readmissions, and mortality) and a strong rationale to study psychological correlates of recovery. Supporting this focus, Takagi et al. [5] synthesized evidence that perioperative depression and anxiety are associated with postoperative mortality in cardiac surgery patients, reinforcing why this population became an early and persistent hotspot. During this stage, burst terms like “recovery” and “adjustment” also point to a conceptual framing of depression as part of the broader psychological adaptation process after major surgery. Derry et al. [46] highlighted the role of preoperative emotional adjustment in temporal lobe epilepsy surgery patients and its association with postoperative depression and adjustment outcomes. Mennander [47] further discussed associations between preoperative depression and postoperative delirium after cardiac surgery, suggesting an early shift toward the practical idea that preoperative screening could inform postoperative management.

### 4.13. Stage 2: Midstage Development (Approximately 2010–2020): Risk Modeling, Predictors, and Clinically Meaningful Outcomes

After 2010, bursts such as “predictors” and “mortality” reflect a transition from describing postoperative distress to building risk stratification frameworks and linking mental health to clinically meaningful endpoints. This shift is consistent with growing recognition that depressive symptoms are not merely transient “stress responses,” but can correlate with complications and long‐term outcomes. Ghoneim and O′Hara [6] summarized associations between depression and postoperative complications, including increased morbidity and mortality and links with infections, cognitive impairment, and chronic pain. Theunissen et al. [48] identified psychosocial status, perioperative pain, and postoperative infection as predictors of psychological outcomes after hysterectomy, and Costelloe et al. [49] described predictors of persistent postoperative pain after spine surgery where depression was among relevant psychological factors. These studies support the interpretation that the field increasingly adopted a prognostic and prehabilitation logic: identifying high‐risk patients preoperatively to tailor perioperative counseling, pain control, and follow‐up.

### 4.14. Stage 3: Recent Frontier (Approximately 2021–2024): High‐Risk Subgroups, Intervention Rigor, and Targeted Prevention

In the most recent period, bursts including “risk,” “elderly patients,” and “double blind” suggest three converging trends. First, “risk” reflects movement toward more granular stratification and targeted preventive strategies, exemplified by Liu and Wang′s [50] analysis in gastric cancer surgery patients, identifying demographic/clinical factors associated with anxiety/depression trajectories and prognosis. Second, “elderly patients” highlights the field′s increasing attention to vulnerable subgroups with higher comorbidity and sensitivity to perioperative stressors. Recent work has reported associations between anesthetic approaches and early postoperative depression/anxiety outcomes in older patients [37], which aligns with an intervention‐relevant question: whether perioperative choices can modify short‐term psychological recovery in high‐risk groups. Third, the burst of “double blind” indicates a methodological push toward more rigorous evaluation of interventions, consistent with the growing intervention literature (e.g., esketamine‐focused syntheses) [44].

### 4.15. Hotspot Clinical Domains and Why They Become “Bursty”

To strengthen the clinical linkage of hotspots mentioned in our burst/timeline discussion, we interpret several recurring surgical domains as follows:

#### 4.15.1. Spine Surgery (Pain–Function–Mood Feedback Loop)

Spine procedures frequently involve chronic pain, functional limitation, and prolonged rehabilitation. Depression can act both as a preoperative vulnerability and a postoperative amplifier of pain perception and disability, which may explain why this area frequently appears in studies linking mood symptoms to recovery trajectories. Longitudinal spine cohort studies have reported associations between depressive symptoms (and related constructs such as life dissatisfaction) and poorer postoperative outcomes over extended follow‐up [33, 34].

#### 4.15.2. Epilepsy Surgery (Neuropsychiatric Comorbidity and Adjustment)

Epilepsy populations have relatively high baseline rates of anxiety/depression, and surgery represents a major life change with uncertain psychosocial adaptation. This makes “adjustment” and mood outcomes clinically salient endpoints. Work in temporal lobe epilepsy surgery has emphasized the importance of preoperative emotional adjustment and its relationship with postoperative depression/adjustment outcomes [46].

#### 4.15.3. Bariatric Surgery (High Baseline Mental Health Burden and Long‐Term Monitoring)

Bariatric patients often have substantial preoperative psychiatric comorbidity and complex postoperative trajectories (behavioral change, body image, and eating behavior), making mental health assessment and follow‐up central. Highly cited syntheses and prospective follow‐up studies in bariatric surgery have described the prevalence and clinical relevance of mental health conditions in these patients and the need for ongoing monitoring [26, 28].

Across these hotspots, the common clinical thread is that postoperative depression is frequently studied where baseline psychiatric comorbidity is common, recovery is prolonged and symptom‐driven, and/or outcomes are sensitive to psychosocial factors.

#### 4.15.4. Future Directions Suggested by the Most Recent Bursts

Based on the recent emphasis on “risk,” “elderly patients,” and more rigorous trial designs, a plausible next step for the field is to combine high‐resolution risk prediction (integrating demographics, comorbidities, perioperative pain, and baseline mental health) with testable intervention packages (e.g., perioperative pharmacologic strategies, structured psychological support, and ERAS‐compatible screening and follow‐up). Additionally, the sustained burst of “prevalence” (2015–2024) indicates that standardized definitions and measurement windows remain important; future studies may benefit from harmonized assessment time points and clearer differentiation between transient postoperative distress and persistent depressive syndromes.

### 4.16. Strengths and Limitations

The strengths of this bibliometric analysis lie in its comprehensive scope and the advanced analytical tools employed. The use of WoSCC provided standardized bibliographic and citation data suitable for bibliometric analysis, allowing consistent extraction of publication, citation, and collaboration information. The application of sophisticated bibliometric tools, including VOSviewer, CiteSpace, and an online R bibliometric platform, enhances the study′s analytical depth. These instruments facilitate a nuanced visualization and interpretation of research trends, leading authors, and collaborative networks, providing a multifaceted view of the field′s development.

However, this study is not without limitations. First, this bibliometric analysis was based solely on the WoSCC. Although WoSCC provides standardized citation data and is widely used for bibliometric studies, relying on a single database may exclude relevant publications indexed in other sources (e.g., Scopus, PubMed, Embase, and PsycINFO), thereby potentially underestimating the volume of research and influencing country/institution/journal rankings and collaboration patterns. Second, a potential language bias is present because only English‐language literature was included, which may overlook important contributions from non–English‐speaking regions and limit the generalizability of “global” comparisons. Third, although we operationally defined postoperative depression as depressive symptomatology occurring after surgery, the construct remains conceptually heterogeneous in the literature. Our broad search strategy may therefore have captured overlapping perioperative psychosocial outcomes rather than a sharply bounded research domain. Accordingly, the keyword and cluster maps should be interpreted cautiously as reflecting the broader perioperative affective and psychosocial literature related to depressive outcomes. Fourth, bibliometric analysis is primarily quantitative and may not fully capture the qualitative depth, clinical heterogeneity, or methodological rigor of individual studies.

Although normalized TC was included as a supplementary time‐adjusted indicator, more advanced year‐normalized or field‐normalized citation indicators were not directly available from the standard WoS export. Therefore, citation visibility was mainly evaluated using TC, average citations, and supplementary normalized TC, consistent with previous bibliometric analyses [51, 52]. Consequently, citation‐based rankings may still be affected by publication year, disciplinary citation practices, and database coverage. Future studies could incorporate more advanced normalized citation metrics and multidatabase retrieval to improve comparability and robustness.

## 5. Conclusions

This bibliometric analysis summarized publication trends, citation patterns, collaboration networks, and research topics in WoSCC‐indexed literature on postoperative depression from 1994 to 2024. The United States, China, and the United Kingdom were the main contributing countries/regions in terms of publication output, whereas several institutions and author groups showed sustained activity within the dataset. Keyword analyses indicated that quality of life, anxiety, symptoms, outcomes, and pain were recurring topics, and recent burst keywords suggested growing attention to risk assessment, elderly patients, prevalence, and double‐blind study designs. These findings provide a descriptive overview of the development of postoperative depression research and may help inform future bibliometric and clinical investigations in this area.

## Author Contributions

Initial conception: Hailong Zhang and Liqin Zhao. Methodological redesign during revision: Ting Li (led) and Liqin Zhao. Administrative support: Hailong Zhang. Initial analysis: Liqin Zhao and Min Li. Reanalysis and standardization during revision: Ting Li (led) and Liqin Zhao. Original draft: Ting Li, Liqin Zhao, Min Li, and Hailong Zhang. Critical revision and rewriting (methods/results) for intellectual content: Ting Li (led), Liqin Zhao, and Hailong Zhang. Ting Li and Liqin Zhao contributed equally to this work.

## Funding

No funding was received for this manuscript.

## Disclosure

All authors approved the manuscript.

## Ethics Statement

The authors have nothing to report.

## Consent

The authors have nothing to report.

## Conflicts of Interest

The authors declare no conflicts of interest.

## Supporting information


**Supporting Information** Additional supporting information can be found online in the Supporting Information section. Table S1: Publication and citation profiles of leading countries. Table S2: Bibliometric indicators of high‐impact journals. Table S3: Publication and citation profiles of high‐impact authors. Table S4: The list of frequently cited publications. Table S5: Keyword clusters (*n* = 153 keywords; seven clusters).

## Data Availability

All data generated or analyzed during this study are included in this published article.
