# Supplementary material for: Mapping Global Trends and Research Hotspots in Postoperative Depression: A Bibliometric Analysis
Source: Behav Neurol. 2026 Aug 6;2026:5578017. doi: 10.1155/bn/5578017 (PMC13447970; doi:10.1155/bn/5578017)
Supplement: Supplementary file 1 [file BN-2026-5578017-s001.docx]

**Supplementary Table 1 Publication and Citation Profiles of Leading Countries**

| **Country** | **Articles** | **Freq** | **SCP** | **MCP** | **MCP_Ratio** | **TP** | **TP_rank** | **TC** | **TC_rank** | **Average Citations** |
| --- | --- | --- | --- | --- | --- | --- | --- | --- | --- | --- |
| USA | 203 | 0.295 | 191 | 12 | 0.059 | 751 | 1 | 6792 | 1 | 33.5 |
| CHINA | 166 | 0.242 | 153 | 13 | 0.078 | 479 | 2 | 1284 | 2 | 7.7 |
| UNITED KINGDOM | 32 | 0.047 | 29 | 3 | 0.094 | 94 | 5 | 1083 | 3 | 33.8 |
| GERMANY | 29 | 0.042 | 26 | 3 | 0.103 | 105 | 4 | 1031 | 4 | 35.6 |
| CANADA | 24 | 0.035 | 20 | 4 | 0.167 | 111 | 3 | 818 | 6 | 34.1 |
| AUSTRALIA | 20 | 0.029 | 18 | 2 | 0.100 | 60 | 9 | 852 | 5 | 42.6 |
| JAPAN | 19 | 0.028 | 19 | 0 | 0.000 | 44 | 10 | 424 | 10 | 22.3 |
| NETHERLANDS | 19 | 0.028 | 12 | 7 | 0.368 | 67 | 7 | 459 | 9 | 24.2 |
| ITALY | 18 | 0.026 | 16 | 2 | 0.111 | 41 | 11 | 467 | 8 | 25.9 |
| FINLAND | 12 | 0.017 | 12 | 0 | 0.000 | 64 | 8 | 545 | 7 | 45.4 |
| KOREA | 12 | 0.017 | 10 | 2 | 0.167 | 30 | 14 | 332 | 12 | 27.7 |
| FRANCE | 11 | 0.016 | 9 | 2 | 0.182 | 74 | 6 | 369 | 11 | 33.5 |
| TURKEY | 11 | 0.016 | 11 | 0 | 0.000 | 34 | 12 | 110 | 19 | 10 |
| ISRAEL | 6 | 0.009 | 4 | 2 | 0.333 | 29 | 16 | 70 | 21 | 11.7 |
| POLAND | 6 | 0.009 | 5 | 1 | 0.167 | 18 | 18 | 144 | 16 | 24 |
| BRAZIL | 5 | 0.007 | 4 | 1 | 0.200 | 24 | 17 | 59 | 23 | 11.8 |
| NORWAY | 5 | 0.007 | 3 | 2 | 0.400 | 31 | 13 | 181 | 14 | 36.2 |
| SWEDEN | 5 | 0.007 | 4 | 1 | 0.200 | 30 | 15 | 80 | 20 | 16 |
| AUSTRIA | 4 | 0.006 | 4 | 0 | 0.000 | 12 | 22 | 122 | 18 | 30.5 |
| BELGIUM | 4 | 0.006 | 4 | 0 | 0.000 | 10 | 26 | 127 | 17 | 31.8 |

Note(s): Articles: Publications of Corresponding Authors only. Freq: Frequence of Total Publications. MCP_Ratio: Proportion of Multiple Country Publications. TP: Total Publications. TP_rank: Rank of Total Publications. TC: Total Citations. TC_rank: Rank of Total Citations. Average Citations: The average number of citations per publication.

**Supplementary Table 2 Bibliometric Indicators of High-Impact Journals**

| **Journal** | **H_index** | **IF** | **JCR_Quartile** | **PY_start** | **TP** | **TP_rank** | **TC** | **TC_rank** |
| --- | --- | --- | --- | --- | --- | --- | --- | --- |
| EPILEPSIA | 10 | 6.6 | Q1 | 1989 | 10 | 2 | 285 | 3 |
| JOURNAL OF PSYCHOSOMATIC RESEARCH | 10 | 3.5 | Q2 | 1977 | 11 | 1 | 287 | 2 |
| PLOS ONE | 8 | 2.9 | Q1 | 2014 | 8 | 8 | 118 | 28 |
| ANESTHESIA AND ANALGESIA | 7 | 4.6 | Q1 | 1991 | 8 | 4 | 176 | 12 |
| EUROPEAN JOURNAL OF ANAESTHESIOLOGY | 7 | 4.2 | Q1 | 1994 | 7 | 9 | 24 | 174 |
| EPILEPSY & BEHAVIOR | 6 | 2.3 | Q2 | 2001 | 8 | 5 | 131 | 24 |
| JOURNAL OF NEUROLOGY NEUROSURGERY AND PSYCHIATRY | 6 | 8.7 | Q1 | 1998 | 7 | 12 | 182 | 11 |
| OBESITY SURGERY | 6 | 2.9 | Q1 | 1998 | 8 | 7 | 159 | 16 |
| SPINE | 6 | 2.6 | Q1 | 2009 | 7 | 13 | 375 | 1 |
| SPINE JOURNAL | 6 | 4.9 | Q1 | 2012 | 7 | 14 | 125 | 25 |
| ANNALS OF THORACIC SURGERY | 5 | 3.6 | Q1 | 1980 | 5 | 20 | 112 | 29 |
| JOURNAL OF AFFECTIVE DISORDERS | 5 | 4.9 | Q1 | 2011 | 7 | 10 | 156 | 18 |
| JOURNAL OF NEUROSURGERY-SPINE | 5 | 2.9 | Q1 | 2014 | 5 | 23 | 106 | 32 |
| WORLD NEUROSURGERY | 5 | 1.9 | Q2 | 2015 | 7 | 15 | 48 | 86 |
| AMERICAN JOURNAL OF GERIATRIC PSYCHIATRY | 4 | 4.4 | Q1 | 2002 | 4 | 28 | 44 | 92 |
| AMERICAN JOURNAL OF TRANSLATIONAL RESEARCH | 4 | 1.7 | Q4 | 2021 | 8 | 3 | 10 | 383 |
| ANNALS OF SURGERY | 4 | 7.5 | Q1 | 2003 | 4 | 29 | 141 | 20 |
| HEART & LUNG | 4 | 2.4 | Q2 | 1991 | 5 | 21 | 57 | 66 |
| JOURNAL OF ARTHROPLASTY | 4 | 3.4 | Q1 | 2018 | 5 | 22 | 189 | 10 |
| JOURNAL OF CRANIOFACIAL SURGERY | 4 | 1 | Q3 | 1997 | 4 | 37 | 18 | 233 |

Note(s): H_index: The h-index of the journal, which measures both the productivity and citation impact of the publications. IF: Impact Factor, indicating the average number of citations to recent articles published in the journal. JCR_Quartile: The quartile ranking of the journal in the Journal Citation Reports, indicating the journal's ranking relative to others in the same field (Q1: top 25%, Q2: 25%-50%, Q3: 50%-75%, Q4: bottom 25%). TP: Total Publications. TP_rank: Rank of Total Publications. TC: Total Citations. TC_rank: Rank of Total Citations. Average Citations: The average number of citations per publication. PY_start: Publication Year Start, indicating the year the journal started publication.

**Supplementary Table 3 Publication and Citation Profiles of High-Impact Authors**

| **Authors** | **H_index** | **g-index** | **m-index** | **PY_start** | **TP** | **TP_Frac** | **TP_rank** | **TC** | **TC_rank** |
| --- | --- | --- | --- | --- | --- | --- | --- | --- | --- |
| AALTO TIMO | 9 | 9 | 0.50 | 2007 | 9 | 1.42 | 1 | 416 | 1 |
| AIRAKSINEN OLAVI | 9 | 9 | 0.50 | 2007 | 9 | 1.42 | 2 | 416 | 1 |
| SINIKALLIO SANNA | 9 | 9 | 0.50 | 2007 | 9 | 1.42 | 3 | 416 | 1 |
| VIINAMAKI HEIMO | 9 | 9 | 0.50 | 2007 | 9 | 1.42 | 4 | 416 | 1 |
| KROGER HEIKKI | 8 | 8 | 0.44 | 2007 | 8 | 1.22 | 5 | 406 | 5 |
| ADOGWA OWOICHO | 5 | 5 | 0.39 | 2012 | 5 | 0.53 | 7 | 207 | 9 |
| LEHTO SOILI M. | 5 | 5 | 0.33 | 2010 | 5 | 0.84 | 8 | 157 | 18 |
| ANTONI MICHAEL H. | 4 | 4 | 0.44 | 2016 | 4 | 0.65 | 9 | 90 | 27 |
| CHENG JOSEPH | 4 | 4 | 0.36 | 2014 | 4 | 0.41 | 11 | 130 | 19 |
| HERNO ARTO | 4 | 4 | 0.22 | 2007 | 4 | 0.58 | 12 | 235 | 8 |
| KEHLET H | 4 | 4 | 0.08 | 1977 | 4 | 0.92 | 14 | 116 | 21 |
| MESSEROTTI BENVENUTI SIMONE | 4 | 4 | 0.31 | 2012 | 4 | 0.79 | 16 | 95 | 24 |
| PALOMBA DANIELA | 4 | 4 | 0.31 | 2012 | 4 | 0.79 | 17 | 95 | 24 |
| PASSCHIER J | 4 | 4 | 0.13 | 1993 | 4 | 0.62 | 19 | 196 | 10 |
| SINGH KERN | 4 | 7 | 0.67 | 2019 | 7 | 0.97 | 6 | 77 | 30 |
| BAGLEY CARLOS A. | 3 | 3 | 0.27 | 2014 | 3 | 0.28 | 23 | 100 | 22 |
| BOEKE S | 3 | 3 | 0.11 | 1997 | 3 | 0.45 | 24 | 179 | 12 |
| CHA ELLIOT D. K. | 3 | 4 | 0.75 | 2021 | 4 | 0.56 | 10 | 48 | 39 |
| DUAN GUANGYOU | 3 | 3 | 0.43 | 2018 | 3 | 0.35 | 27 | 24 | 48 |
| DUITS AA | 3 | 3 | 0.11 | 1997 | 3 | 0.45 | 28 | 179 | 12 |

Note(s): H_index: The h-index of the journal, which measures both the productivity and citation impact of the publications. g_index: The g-index of the journal, which gives more weight to highly-cited articles. m_index: The m-index of the journal, which is the h-index divided by the number of years since the first published paper. TP: Total Publications. TP_rank: Rank of Total Publications. TC: Total Citations. TC_rank: Rank of Total Citations. Average Citations: The average number of citations per publication. PY_start: Publication Year Start, indicating the year the journal started publication.

**Supplementary Table 4 The list of frequently cited publications**

| **Publications** | **DOI** | **Total Citations** | **TC per Year** | **Normalized TC** |
| --- | --- | --- | --- | --- |
| DAWES AJ, 2016, JAMA-J AM MED ASSOC | 10.1001/jama.2015.18118 | 345 | 38.33 | 8.00 |
| VOON V, 2008, BRAIN | 10.1093/brain/awn214 | 324 | 19.06 | 5.87 |
| DE ZWAAN M, 2011, J AFFECT DISORDERS | 10.1016/j.jad.2011.03.025 | 265 | 18.93 | 4.36 |
| TAENZER P, 1986, PAIN | 10.1016/0304-3959(86)90119-3 | 250 | 6.41 | 2.62 |
| LITOFSKY NS, 2004, NEUROSURGERY | 10.1227/01.NEU.0000103450.94724.A2 | 202 | 9.62 | 3.42 |
| RAEBEL MA, 2013, JAMA-J AM MED ASSOC | 10.1001/jama.2013.278344 | 186 | 15.50 | 4.22 |
| DEVINSKY O, 2005, NEUROLOGY | 10.1212/01.wnl.0000187114.71524.c3 | 173 | 8.65 | 2.24 |
| MUNAFÒ MR, 2001, J PSYCHOSOM RES | 10.1016/S0022-3999(01)00258-6 | 173 | 7.21 | 2.95 |
| HO B, 2016, FOOT ANKLE INT | 10.1177/1071100716665113 | 166 | 18.44 | 3.85 |
| KUDOH A, 2002, ANESTH ANALG | 10.1097/00000539-200207000-00020 | 162 | 7.04 | 2.85 |
| ÖZALP G, 2003, ACTA ANAESTH SCAND | 10.1034/j.1399-6576.2003.470105.x | 155 | 7.05 | 2.39 |
| PAPAIOANNOU M, 2009, PAIN MED | 10.1111/j.1526-4637.2009.00730.x | 143 | 8.94 | 4.49 |
| DANIELE A, 2003, J NEUROL NEUROSUR PS | 10.1136/jnnp.74.2.175 | 142 | 6.45 | 2.19 |
| CARROLL I, 2012, ANESTH ANALG | 10.1213/ANE.0b013e31825c049f | 139 | 10.69 | 3.33 |
| ZARGOOSHI J, 2001, J UROLOGY | 10.1016/S0022-5347(05)65677-7 | 139 | 5.79 | 2.37 |
| WANG J, 2011, ANESTHESIOLOGY | 10.1097/ALN.0b013e31822f16ae | 138 | 9.86 | 2.27 |
| PONS TP, 1992, J NEUROPHYSIOL | 10.1152/jn.1992.68.2.518 | 134 | 4.06 | 3.94 |
| GILLIAM F, 1999, NEUROLOGY | 10.1212/WNL.53.4.687 | 129 | 4.96 | 1.95 |
| WELTER ML, 2014, NEUROLOGY | 10.1212/WNL.0000000000000315 | 124 | 11.27 | 4.46 |
| ASTOR MC, 2016, J CLIN ENDOCR METAB | 10.1210/jc.2016-1477 | 118 | 13.11 | 2.74 |
| SINIKALLIO S, 2007, EUR SPINE J | 10.1007/s00586-007-0349-3 | 116 | 6.44 | 2.11 |
| TULLY PJ, 2008, J BEHAV MED | 10.1007/s10865-008-9153-8 | 115 | 6.76 | 2.08 |
| ROTH ML, 2007, PAIN RES MANAG | 10.1155/2007/394960 | 114 | 6.33 | 2.08 |
| MORTERO RF, 2001, ANESTH ANALG |  | 110 | 4.58 | 1.88 |
| RYMASZEWSKA J, 2003, EUR PSYCHIAT | 10.1016/S0924-9338(03)00052-X | 106 | 4.82 | 1.63 |
| PIRRAGLIA PA, 1999, INT J GERIATR PSYCH | 3.0.CO;2-9" target="_blank">10.1002/(SICI)1099-1166(199908)14:83.0.CO;2-9 | 106 | 4.08 | 1.6 |
| DUITS AA, 1997, PSYCHOSOM MED | 10.1097/00006842-199705000-00009 | 106 | 3.79 | 2.11 |
| UCHIDA A, 1982, JNCI-J NATL CANCER I |  | 103 | 2.4 | 3.22 |
| SINIKALLIO S, 2011, SPINE | 10.1097/BRS.0b013e3181dcaf4a | 102 | 7.29 | 1.68 |
| ROYSE C, 2003, ANN THORAC SURG | 10.1016/S0003-4975(02)04074-2 | 101 | 4.59 | 1.56 |
| RILEY J, 2012, NEUROSURGERY | 10.1227/NEU.0b013e31825ca05f | 97 | 7.46 | 2.33 |
| LEUNG JM, 2005, J GERONTOL A-BIOL | 10.1093/gerona/60.12.1563 | 96 | 4.8 | 1.24 |
| SOULAS T, 2008, J NEUROL NEUROSUR PS | 10.1136/jnnp.2007.130583 | 95 | 5.59 | 1.72 |
| CUNNINGHAM SJ, 1996, BRIT J ORAL MAX SURG | 10.1016/S0266-4356(96)90271-5 | 95 | 3.28 | 3.41 |
| JAMISON RN, 1993, ANESTH ANALG |  | 95 | 2.97 | 2.03 |
| CLARK WC, 2002, PAIN |  | 93 | 4.04 | 1.63 |
| WRENCH J, 2004, EPILEPSIA | 10.1111/j.0013-9580.2004.48803.x | 92 | 4.38 | 1.56 |
| ALTSHULER L, 1999, J NEUROPSYCH CLIN N | 10.1176/jnp.11.4.436 | 91 | 3.5 | 1.37 |
| ALTHAUS A, 2014, EUR J PAIN | 10.1002/j.1532-2149.2013.00385.x | 89 | 8.09 | 3.2 |
| MAIER F, 2013, J NEUROL NEUROSUR PS | 10.1136/jnnp-2012-303670 | 89 | 7.42 | 2.02 |
| ANDREW MJ, 2000, J PSYCHOSOM RES | 10.1016/S0022-3999(00)00089-1 | 89 | 3.56 | 2.2 |
| MACFIE J, 2000, NUTRITION | 10.1016/S0899-9007(00)00377-4 | 88 | 3.52 | 2.17 |
| CHO CH, 2015, CLIN ORTHOP RELAT R | 10.1007/s11999-015-4258-1 | 86 | 8.6 | 3.08 |
| LAM WWT, 2013, J CLIN ONCOL | 10.1200/JCO.2012.45.1856 | 86 | 7.17 | 1.95 |
| KEMPH JP, 1966, AM J PSYCHIAT | 10.1176/ajp.122.11.1270 | 86 | 1.46 | 1.76 |
| WEBER T, 2013, JAMA SURG | 10.1001/2013.jamasurg.316 | 85 | 7.08 | 1.93 |
| SHULDHAM CM, 2002, EUR HEART J | 10.1053/euhj.2001.2897 | 85 | 3.7 | 1.49 |
| STANLEY BM, 1998, AUST NZ J SURG | 10.1111/j.1445-2197.1998.tb04678.x | 85 | 3.15 | 1.84 |
| GOYAL TM, 2005, PSYCHOSOM MED | 10.1097/01.psy.0000174046.40566.80 | 79 | 3.95 | 1.02 |
| MILLER JA, 2015, SPINE J | 10.1016/j.spinee.2014.06.020 | 77 | 7.7 | 2.75 |

**Supplementary Table 5. Keyword clusters (n = 153 keywords; 7 clusters)**

| Cluster | No. of keywords | Keywords |
| --- | --- | --- |
| Cluster 1 | 25 | adjustment; age; analgesia; brain; cancer; cancer-patients; depression; elderly-patients; enhanced recovery; follow-up; general-anesthesia; impairment; lobe epilepsy; lobectomy; memory; morphine; performance; postoperative pain; primary-care; resection; sleep; stroke; surgery; surgical-treatment; temporal-lobe epilepsy |
| Cluster 2 | 25 | artery-bypass-surgery; cardiac-surgery; coronary-artery-disease; determinants; disease; epidemiology; gender-differences; graft-surgery; health-status; heart-disease; heart-surgery; morbidity; mortality; myocardial-infarction; of-life; patterns; population; prognosis; recovery; risk; risk-factor; social support; stenosis; survival; symptoms |
| Cluster 3 | 24 | antidepressant; augmentation; bypass graft-surgery; decompression; diagnosis; disability index; disorder; efficacy; injury; inventory; low-back-pain; management; metaanalysis; outcomes; pain; predictors; readmission; rehabilitation; repair; scale; spinal stenosis; surgical-management; therapy; united-states |
| Cluster 4 | 21 | anxiety; arthroplasty; breast-cancer; c-reactive protein; coronary-heart-disease; cytokines; expression; fatigue; inflammation; intervention; ketamine; major depression; mechanisms; mood; posttraumatic-stress-; psychological distress; randomized controlled; stress; survivors; total hip-replacement; women |
| Cluster 5 | 20 | anesthesia; chronic pain; deep-brain-stimulation; delirium; disorders; double-blind; dysfunction; impact; knee arthroplasty; life; model; multicenter; older-adults; prevention; quality; responses; satisfaction; score; severity; trial |
| Cluster 6 | 19 | behavior; complications; depression scale; disability; experience; gender; health survey; hospital anxiety; osteoarthritis; patient; preoperative anxiety; psychological-factors; questionnaire; reliability; replacement; subthalamic nucleus s; total hip; validation; validity |
| Cluster 7 | 19 | adults; association; care; comorbidity; dementia; depressive symptoms; distress; gastric bypass-surger; health; mental-health; obesity; phq-9; prevalence; psychiatric-disorders; psychometric propert; quality-of-life; reported outcomes; risk-factors; weight-loss |
